# Why the gaze behavior of expert physicians and novice medical students differ during a simulated medical interview: A mixed methods study

**DOI:** 10.1371/journal.pone.0315405

**Published:** 2025-01-02

**Authors:** Rie Yamada, Kuangzhe Xu, Satoshi Kondo, Makoto Fujimoto

**Affiliations:** 1 Department of Adult Nursing, Faculty of Medicine, Academic Assembly, University of Toyama, Toyama, Japan; 2 Institute for Promotion of Higher Education, Hirosaki University, Aomori, Japan; 3 Department of Medical Education, Graduate School of Medicine, University of Toyama, Toyama, Japan; 4 Center for Medical Education and Career Development, Graduate School of Medicine, University of Toyama, Toyama, Japan; 5 Department of Japanese Oriental Medicine, Faculty of Medicine, Academic Assembly, University of Toyama, Toyama, Japan; Zeiss Group: Carl Zeiss AG, GERMANY

## Abstract

Human cognition is reflected in gaze behavior, which involves eye movements to fixate or shift focus between areas. In natural interactions, gaze behavior serves two functions: signal transmission and information gathering. While expert gaze as a tool for gathering information has been studied, its underlying cognitive processes remain insufficiently explored. This study investigated differences in gaze behavior and cognition between expert physicians and novice medical students during a simulated medical interview with a simulated patient, drawing implications for medical education. This study employed an exploratory sequential mixed methods design. During the simulated medical interview, participants’ gaze behavior was measured across five areas: the patient’s eyes, face, body trunk, medical chart, and medical questionnaire. A hierarchical Bayesian model analyzed differences in gaze behavior between expert physicians and novice medical students. Then, a semi-structured interview was conducted with participants to discern their perceptions during their gaze behavior; their recorded gaze behavior was presented to them, and analyzed using a qualitative descriptive approach. Model analyses indicated that experts looked at the simulated patient’s eyes less frequently compared to novices during the simulated medical interview. Expert physicians stated that because of the potential for discomfort, looking at the patient’s eyes was less frequent, despite its importance for obtaining diagnostic findings. Conversely, novice medical students did not provide narratives for obtaining such findings, but increased the number of times they did so to improve patient satisfaction. This association between different perceptions of gaze behavior may lead to new approaches in medical education. This study highlights the importance of understanding gaze behavior in the context of medical education and suggests that different motivations underlie the gaze behavior of expert physicians and novice medical students. Incorporating training in effective gaze behavior may improve the quality of patient care and medical students’ learning outcomes.

## Introduction

The medical interview is a critical component of the diagnostic process, contributing to approximately 80% of diagnoses [1, 2]. Within this context, a physician’s gaze behavior—defined as the act of directing eye movements to fixate on specific regions or shift focus from one location to another [3]—plays a crucial role in both building trust with the patient [4] and gathering the essential information needed for accurate diagnosis and treatment [5]. In natural face-to-face interactions, gaze behavior serves a dual function: it signals transmission social interest and communicative intent and enables individuals to observe the eyes and face of others, interpreting their focus of attention and behavioral intentions [6, 7]. Gaze behavior plays a critical role in both signal transmission and information gathering [8]. However, traditional studies conducted in laboratory settings have primarily relied on images or videos, which may not fully capture the dual function of gaze behavior in real-world, face-to-face interactions [8]. To address this gap, recent advances have employed dual eye-tracking technology to investigate gaze behavior in more naturalistic, face-to-face scenarios.

Dual eye-tracking is a technique that records and analyzes the gaze behavior of both individuals during face-to-face interactions, allowing for detailed examination of interaction dynamics, such as mutual gaze and gaze synchronization [8]. For instance, in natural settings, mutual gaze during face-to-face interactions only occurs approximately 10% of the time [9], yet the information exchanged during these brief moments of eye contact plays a crucial role in building trust and promoting cooperative behavior [10]. Further, even gaze-anxious individuals tend to exhibit typical gaze behavior in naturalistic face-to-face settings to maintain interaction [11]. These findings provide key insights into the fundamental mechanisms of interpersonal interaction in natural settings, offering valuable predictors for behavioral and psychological outcomes [12, 13].

In contrast to typical social interactions, a medical interview has a distinct purpose: to effectively gather diagnostic and therapeutic information while simultaneously building rapport with the patient, all within the constraints of limited time. Direct gaze during short interactions is often associated with positive evaluations and perceptions of warmth and trustworthiness from the other party [14]. Therefore, physicians’ gaze behavior is likely more intentional and goal oriented. It involves two key strategies: signal transmission trust and rapport through well-timed eye contact, and efficiently gathering diagnostic information necessary for clinical decision-making. Physicians’ gaze behavior develops through experience, enhancing both diagnostic efficiency and accuracy [15]. Therefore, studies using eye trackers have examined the gaze behavior of expert physicians (experts), demonstrating their ability to quickly identify areas relevant to diagnosis. For example, in studies on pathological image interpretation [15–17], electrocardiogram analysis [18], and simulator-based treatments [9, 10], experts’ gaze was not dispersed [18–20], and the number and duration of fixations on specific areas were fewer compared to novice medical students (novices) [15–17]. However, most of these studies focused on information gathering during diagnostic imaging, such as X-rays or MRIs, or during treatments like surgery or anesthesia. The dual function of experts’ gaze behavior during a medical interview—signal transmission and information gathering—has not been sufficiently explored.

Previous research on physicians’ gaze behavior during a medical interview has typically relied on video recordings for analysis. Studies have reported that the frequency of eye contact between physicians and patients is associated with greater patient self-disclosure [21, 22], higher patient satisfaction [23], and a more patient-centered approach to care [24]. However, these studies did not use eye-tracking technology to measure gaze behavior, limiting the precision of the quantitative evaluation of eye contact. Human eye movements are subtle, and without the use of eye trackers, it is difficult to accurately determine where a person is looking [25]. Therefore, an accurate quantitative assessment of physicians’ gaze behavior during a medical interview using eye-tracking technology is necessary.

As previously mentioned, experts’ gaze behavior during a medical interview is suspected to involve fewer fixations and shorter fixation durations on specific areas compared to novices. Gaze behavior is closely related to cognitive processes such as attention, memory [26, 27], and decision-making [28]. Therefore, the differences in gaze behavior between experts and novices during a medical interview are likely related to underlying cognitive differences, which may relate to the dual function of gaze behavior in signal transmission and information gathering. However, these differences have not yet been thoroughly explored.

This study addresses two research questions related to the gaze behavior of experts and novices during a medical interview and the cognition underlying this behavior: 1) How does gaze behavior toward patients differ between expert physicians and novice medical students? 2) How does cognition related to gaze behavior toward patients differ between expert physicians and novice medical students? To answer these questions, we decided to use an eye-tracker [18], which is effective for investigating the complex interactions between gaze behavior and cognition, in an actual outpatient clinic setting rather than a laboratory environment.

Further, our previous study [29] indicated that specialist physicians, during simulated consultations, focused their gaze on five key areas: the simulated patient’s (SP) eyes, face, body trunk, medical chart, and medical questionnaire. We adopted the same five points of fixation, expecting that this approach would allow for a detailed comparison of the differences in gaze behavior and the underlying cognition between experts and novices.

The study objectives are as follows: a) to quantitatively assess the gaze behavior of experts and novices during interactions with a SP and explore the differences in gaze behavior between the two groups; and b) to qualitatively describe the cognition underlying the gaze behavior of experts and novices toward a SP and investigate the cognitive differences between the two groups. Through this study, we aim to transform the tacit experiential knowledge related to the dual functions of experts’ gaze behavior—signal transmission and information gathering—into explicit knowledge that can be communicated and shared, with the goal of applying it to both medical education and the education of gaze behavior.

## Materials and methods

### Research paradigm and design

Understanding phenomena demands appropriate methodology, without the limitation of a qualitative or quantitative approach, as the research questions demand. Therefore, pragmatism and a research paradigm that integrated qualitative and quantitative approach [30] were employed.

This study used an exploratory sequential mixed methods design, comprising two distinct phases: quantitative research, followed by qualitative research. In the first phase, researchers collected and analyzed quantitative data [31]. In the next phase, they collected and analyzed qualitative data as it helps to explain or build on the findings of the first phase’s quantitative research [31]. The second phase’s qualitative data builds on the first phase’s quantitative findings, and the two phases are linked in the intermediate stages of the research [31]. The theoretical rationale for this approach is that quantitative data and the associated qualitative analyses provide a general understanding of the research questions [30]. Therefore, to understand the complex phenomenon of cognitive processes in the gaze behavior of experts and novices, this study used an exploratory sequential design, which can provide more valuable insights into a phenomenon and, cannot be fully understood using a single research design [32].

In this study’s first phase of quantitative analyses, a wearable eye-tracker was used to measure and quantitatively evaluate the gaze behavior of experts and novices during a simulated medical interview and identify differences in their respective gaze behavior.

**Fig 1:**
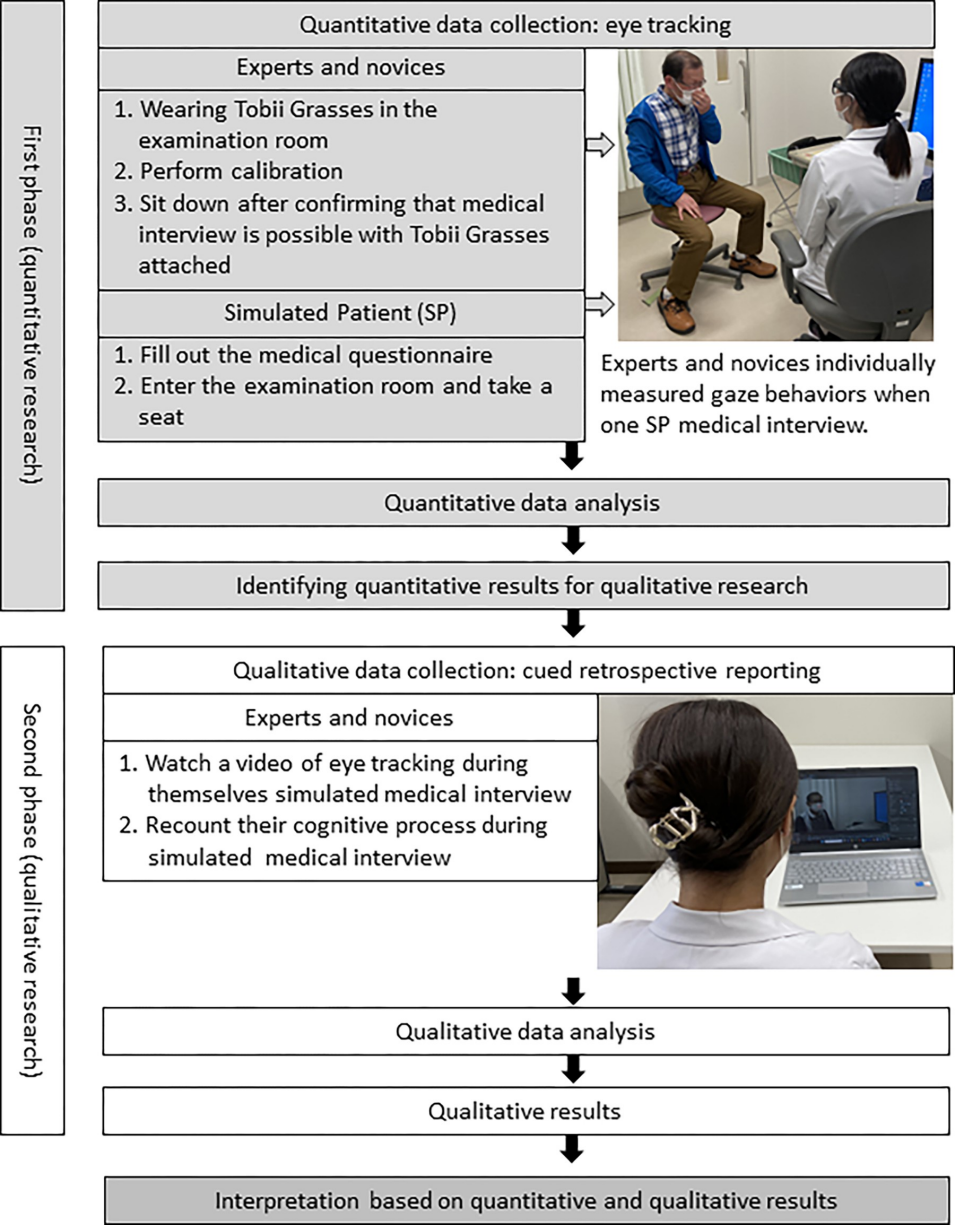
Overview of the explanatory sequential design.

During the second phase, to explore the implications of differences in gaze behavior, qualitative data were collected through cued retrospective reporting, in which experts and novices were presented with their gaze measurement data and asked to recall and verbalize their cognitive processes during gaze behavior. Their data were then analyzed using a qualitative descriptive approach. This study was conducted in Japan. Data was collected from February 2022 to August 2023. Fig 1 shows an overview of this study’s mixed methods research design.

### Ethical considerations

This study was approved by the Research Ethics Committee of the University of Toyama (No. R2021056) and conducted in accordance with the guidelines and regulations of the Declaration of Helsinki. The researcher explained in writing and orally to the experts, novices, and the SP the study’s main purpose, the voluntary nature of their participation, their freedom to discontinue without any disadvantage, the confidentiality of their personal information, that the results may be published, and that their data would be destroyed at the end of the study. Participants’ written informed consent to participate and to publish their images in an online, open-access publication was obtained. Further, the participants provided written informed consent (as outlined in the *PLOS ONE* consent form) for these case details to be published.

### Research team and reflexivity

The research team comprised two medical education researchers with more than 10 years of experience as clinicians (MF and SK), a nurse and health sciences researcher (RY), and a cognition researcher (KX). RY and SK had experience of conducting and publishing qualitative research in a constructivist paradigm. KX and MF had extensive experience of quantitative research and eye measurements. All members had research experience in medical education.

RY and SK, who led the qualitative research did not know the experts and novices and were not involved in evaluating the novices’ performance. To establish a relationship with the experts and approach the study with a general understanding of their views, attitudes, and behavior, RY attended their seminars and workshops and participated in their medical examination a year before study commencement.

### Participants

To qualify as an expert requires a level of high performance with a wealth of skills and knowledge, and at least 10 years of experience [33, 34]. Therefore, this study’s eligibility criteria were defined as follows: for experts, a minimum of 10 years of experience as a physician; for novices, medical students in their 5th or 6th year who had passed the Computer Based Test and the Pre-Clinical Clerkship Objective Structured Clinical Examination (OSCE).

Participants were recruited by 1) soliciting participation from physicians at Toyama University Hospital and medical students at Toyama University Faculty of Medicine, and 2) snowball sampling from among experts and novices who agreed to participate. Research cooperation was obtained from eight experts (seven men and one woman; mean age 51.9 ± 8.1 years; mean years of experience as physicians 26.5 ± 8.7 years) and nine novices (four men and five women; mean age 27.6 ± 6.0 years).

Six of the eight experts and seven of the nine novices, all without eye diseases aside from refractive errors, used vision correction through glasses or contact lenses.

To recruit an SP, research cooperation was requested from volunteers registered at the Centre for Older People. A man in his early 70s, who agreed to participate, and did not know either the experts or the novices, was selected as an SP. He was diagnosed with benign prostatic hyperplasia 20 years ago, took one tablet of 0.2 mg tamsulosin hydrochloride daily, and experienced nocturia 2–3 times/night.

The novices and the SP were paid a small remuneration.

### First phase: Quantitative research

#### Simulated medical interview’s environment and setting

The data collection site was the outpatient examination room of Toyama University Hospital. The experts/novices and SP sat on chairs (height: 45 cm) placed approximately 115 cm apart. The room temperature was set at 26.0 ± 2.0°C.

This setting was where experts conducted their daily clinical practice, and novices had already performed an initial patient interview as part of their clinical training. The environment of the simulated medical interview closely resembled their usual clinical practice setting.

During the simulated medical interview, the experts and novices were asked to obtain the SP’s medical information relating to chief medical complaints, current and past medical history, and family history, and record this information in the medical records. They were also told that it was up to them when to review the medical questionnaire. No time limit was set for the simulated medical interview. The SP’s diagnoses and prescribed medications were not given during the expert and novice sessions.

#### Wearable eye tracker

This study used Tobii Pro Glasses 3, a 50-Hz wearable wireless eye-tracker (Tobii Technology, Danderyd Municipality, Sweden), a glasses-type gaze-measuring system called the head unit. It included Tobii Glasses Controller software (version 1.141) for recording gaze, and Tobii Pro Lab (version 1.194) analysis software. The Tobii Pro Glasses feature a high-resolution camera embedded in the forehead area that records the wearer’s gaze and visual field, enabling the distinction and analysis of the five gaze behavior types described below. Fixations were defined as periods during which the velocity of eye movements dropped below the Tobii Pro I-VT filter threshold (30 degrees/second) and lasted at least 200 ms.

#### Measurement items

Referring to the classification of gaze behavior in interpersonal relationships by Cranach [35], the gaze behavior of experts and novices toward the SP’s face was categorized as follows: eye gaze (EG) and face gaze (FG). Based on previous studies on expert physicians’ gaze behavior during a simulated medical interview [29], gaze behavior toward the SP’s body and other objects was categorized as follows: body trunk gaze (BG), medical chart gaze (MG), and medical questionnaire gaze (MQG). Table 1 defines these five gaze behavior types. While this study primarily focused on the EG of experts and novices, it also analyzed other gaze behavior, hoping to observe significant differences in them as well.

**Table 1:** Start and end points of the five gaze behavior types among experts and novices.

Gaze behavior	Start point	End point
Eye gaze (EG)	The point when they gaze into the SP’s eyes	The point when they gaze at something, other than the SP’s eyes
Face gaze (FG)	The point when they gaze at a face, other than the SP’s eyes	The point when they gaze at something, other than the SP’s face
Body trunk gaze (BG)	The point when they gaze at the SP’s body trunk	The point when they gaze at something, other than the SP’s body trunk
Medical chart gaze (MG)	The point when they gaze at the medical chart	The point when they gaze at something, other than the medical chart
Medical questionnaire gaze (MQG)	The point when they gaze at the medical questionnaire	The point when they gaze at something, other than the medical questionnaire

**Fig 2:**
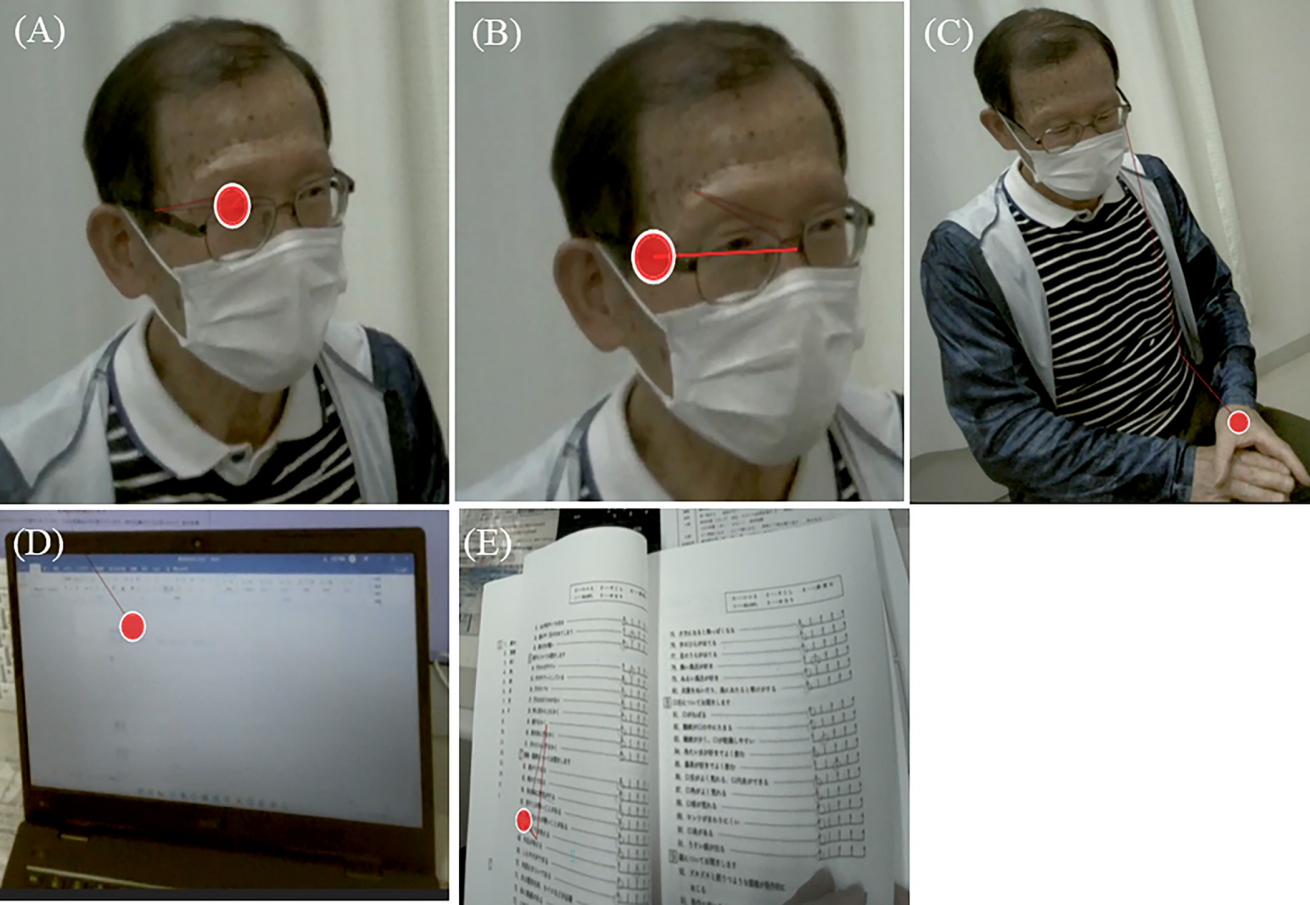
Five gaze behavior types among experts and novices. (A), Eye gaze: one-sided gaze at the SP’s eyes. (B), Face gaze: one-sided gaze at the SP’s face, other than the eyes. (C), Body trunk gaze: gaze at the SP’s body trunk. (D), Medical chart gaze: gaze at the medical chart. (E), Medical questionnaire gaze: gaze at the medical questionnaire. The red circles on the images indicate the point of gaze of experts and novices. The red lines on the images indicate the trajectory of gaze behavior.

Several previous studies on gaze analysis used total gaze duration as a measurement item [36], whereas this study additionally defined the start and end points of the five gaze behavior types to measure fixation duration. Fig 2 defines the five gaze behavior types.

#### Simulated medical interview procedure

Prior to the medical interview, the SP was asked to complete a questionnaire on sleep, appetite, defecation, and general condition. Experts and novices who did not require vision correction wore the Tobii Pro Glasses 3 without any corrective lenses and underwent the eye-tracking measurement with their natural vision. However, experts and novices who required vision correction removed their glasses and attached prescription lenses, which can be magnetically fitted to the Tobii Pro Glasses 3, to correct their vision before undergoing the eye-tracking measurement. The researchers then used Tobii Glasses Controller software to calibrate the gaze control behavior of the experts and novices, who were asked to sit in a chair wearing Tobii Pro Glasses. The batteries supplied with the Tobii Pro Glasses were placed in the pocket of their examination gowns to ensure no interference with the medical interview. The SP waiting in the outpatient waiting room entered the examination room and sat on a chair. The gaze behavior of experts and novices during the medical interview of one SP, were recorded with Tobii Pro Glasses.

### Statistical analysis

#### Data processing

We conducted the coding based on the manual coding method by Yamamoto et al. [37]. Specifically, the first author (RY) used the Tobii Pro Lab Analyzer software version 1.232 (Tobii Technology) to review each video frame recorded from the perspectives of experts and novices. The videos were played back at 1/16 speed and, based on the five gaze behavior types previously described (Table 1), the start and end points of each gaze behavior were coded. The fourth author (MF) independently assessed 20% of the gaze behavior randomly selected from all the data. The interrater reliability between the first and fourth authors was calculated using the kappa coefficient with IBM SPSS Statistics version 28 (IBM Corp., Armonk, NY, USA), resulting in a kappa coefficient of 0.98, indicating a high level of agreement.

After manually coding the start and end points using Tobii Pro Lab Analyzer software (Tobii Technology), the data were exported in Excel format. From the start and end points in the Excel data, we calculated the fixation times for each of the five gaze behavior types for both experts and novices. This enabled us to compare and analyze the fixation times between the two groups.

During the medical interview, gaze behavior toward each facial region was converted into the percentage of time spent focusing on each region relative to the total interview duration. Specifically, each participant’s gaze behavior on each facial region was quantified as a percentage by dividing the fixation time on each region by the total duration of the medical interview. For instance, if the recorded fixation time for EG was 250 ms (30 Hz) and the duration of the medical interview was 50 000 ms (6000 Hz), the resulting percentage for EG would be 0.005. The fixation counts for the medical interview were as follows: experts,—30 263.25 ± 16 064.31; novices,—32 028.11 ± 21 709.25.

#### Model building

**Fig 3:**
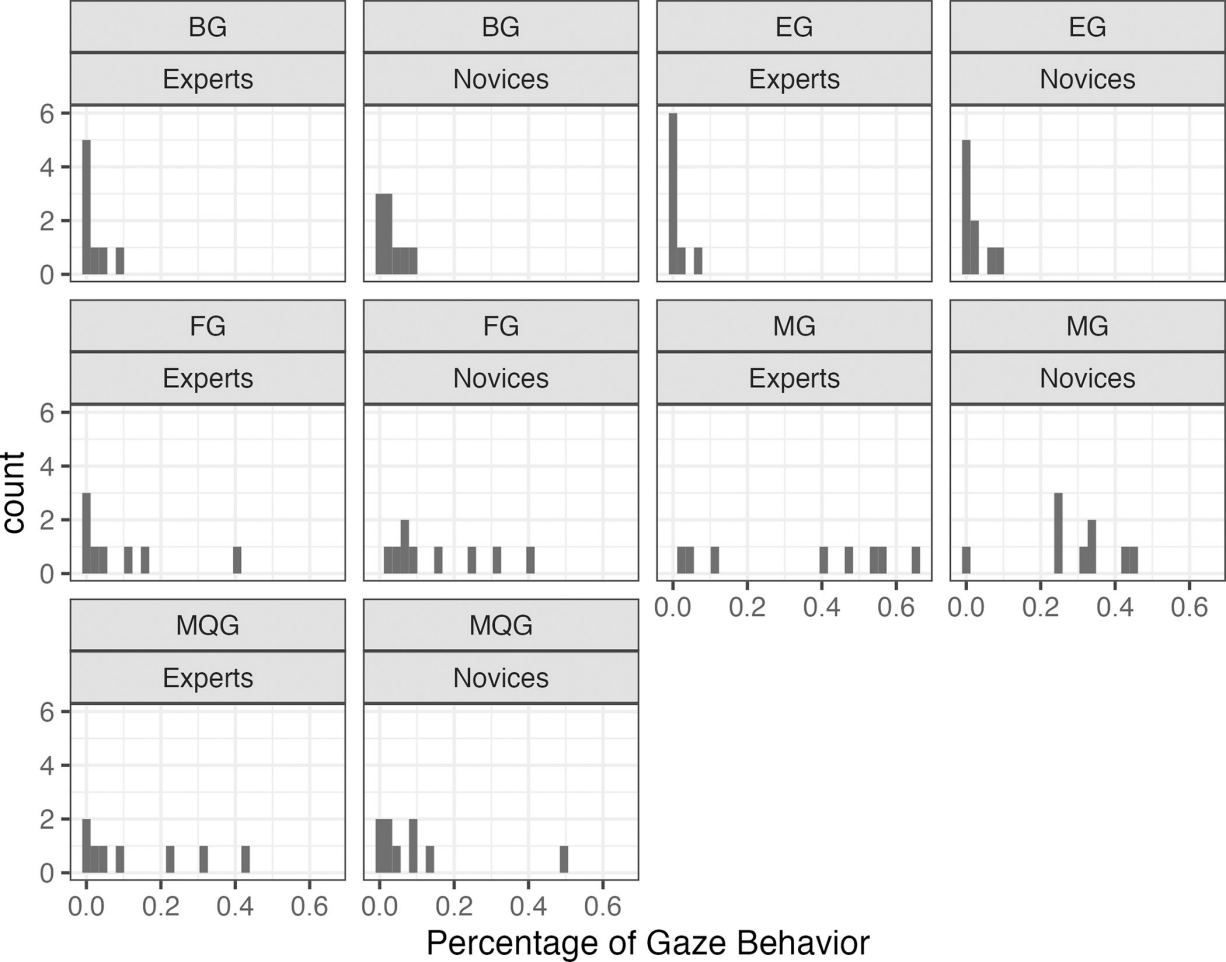
Distribution of percentages of gaze behavior among experts and novices during the medical interview. The x-axis represents the percentage of gaze behavior, while the y-axis indicates the count of occurrences for each percentage.

ZIB (Fig 3) is a mixed distribution, incorporating “0” data, and bounded within the range of 0–1. It combines a Bernoulli distribution, which estimates whether the value is “0;” and a beta distribution, which models the distribution for values other than “0.” By utilizing this characteristic, the probability of “gaze behavior toward a specific part in a specific scenario” and “the frequency of gaze behavior directed toward a specific part in a specific scenario,” were predicted.

Referring to previous studies [38–40], this study constructed a ZIB model, expressed in Eqs (1)–(6) below. The model elucidates the relationship between novices and experts regarding specific gaze behavior (G_k, where k represents indices for the following gaze behavior: EG, FG, BG, MG, and MQG). The parameter q_k is used to estimate the Bernoulli distribution, and its relationship with the examinees’ characteristics is expressed in Eq (3). Additionally, parameters a_k and b_k are used to estimate the beta distribution, and their relationships with the examinees’ characteristics are expressed in Eqs (4)–(6). The parameters for the beta distribution are detailed in Eqs (4) and (5). The model’s linear components illustrate that whether a participant is a novice or a expert (V_k) is associated with whether a specific area is observed (Eq [3]) and the extent of observation (Eq [6]). These were treated as fixed effects (β_k^bern for Bernoulli and β_k^beta for beta), while r^subj represents the random effects attributable to participants.


$$G_{k}∼ZIB(q_{k},a_{k},b_{k})$$ (1)

$$ZIB(G_{k}|q_{k},a_{k},b_{k})=\{\begin{array}{l} Bern(0|q_{k})\;(G_{k}=0)\\ Bern(1|q_{k})\times Beta(G_{k}|a_{k},b_{k})(G_{k}>0) \end{array}$$ (2)

$$q_{k}=\frac{1}{1+exp({-(\alpha }_{k}^{bern}+\beta_{k}^{bern}V_{k}+r^{subj}))}$$ (3)

$$a_{k}=\phi \cdot \mu_{k}$$ (4)

$$b_{k}=\phi (1-\mu_{k})$$ (5)

$$\mu_{k}=\frac{1}{1+exp({-(\alpha }_{k}^{beta}+\beta_{k}^{beta}V_{k}+r^{subj}))}$$ (6)

The Rstan package [41–43] was used for parameter estimation. The prior distribution for fixed effects was a normal distribution with a mean of 0 and an SD of 50. Default Stan hyperparameters (chain = 4, thin = 1, iteration = 2000, and warm-up = 1000) were used. Convergence was assessed using Rhat (R) for each parameter, with convergence criteria of at least 3 chains, and Rhat < 1.1 for all parameters. All parameter estimations met the convergence criteria. Additionally, the highest density interval (HDI) was used to determine parameter significance, considering effects as “significant” when the HDI did not include “0.”

#### Quantitative results

The simulated medical interview times were as follows—experts, 10.0159 ± 4.916 minutes; novices, 11.2426 ± 7.0123 minutes—with no significant difference observed.

**Fig 4:**
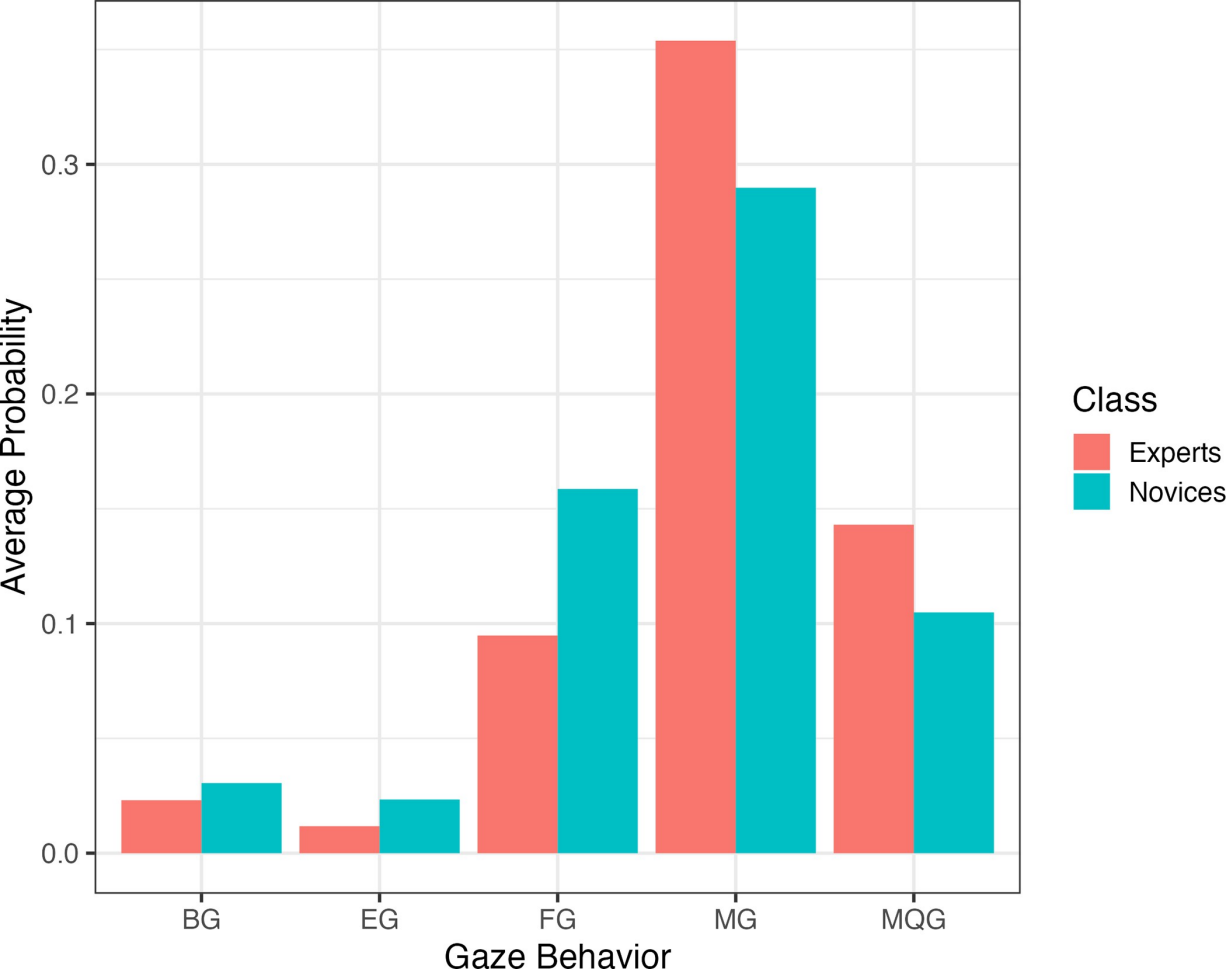
Average gaze behavior percentage of experts and novices. The x-axis represents the percentage of gaze behavior, while the y-axis indicates the count of occurrences for each percentage.

Fig 4 shows the percentages of average gaze behavior of experts and novices. Experts exhibited higher occurrences of MG and MQG than novices, who displayed higher frequencies of FG. Gaze behavior was relatively infrequent in both the BG and EG groups.

Table 2 presents the results of analyses using a ZIB regression model to detect significant differences in gaze behavior between experts and novices during the medical interview. If the 95% HDI does not include “0,” the effect of the gaze behavior is considered “significant.” If the mean is positive, it indicates that novices are observed more frequently, whereas a negative mean suggests that experts are observed more frequently. This analysis confirmed that novices tend to focus more on the SP’s eyes (EG) compared to experts (mean = 30.533; 95% HDI: 0.702–70.703). There were no significant differences between experts and novices in FG, BG, MG, and MQG.

**Table 2:** Comparing gaze behavior of experts and novices in the simulated medical interview (zero-inflated beta distribution).

Pattern	Gaze behavior	Mean	95% HDI^a^	
			Lower limit	Upper limit
Bernoulli	**EG**	**30.533**	**0.702**	**70.703**
	FG	37.425	-24.960	107.456
	BG	-17.413	-45.609	2.079
	MG	-18.117	-44.675	2.251
	MQG	28.523	-1.609	70.155
Beta	EG	0.360	-1.060	1.801
	FG	1.012	-0.307	2.486
	BG	0.523	-0.555	1.763
	MG	0.106	-0.914	-1.288
	MQG	-0.630	-2.070	0.725

^a^If “0” is not included in the lower and upper limits of the 95% highest density interval, it is considered significant. **Bold** areas indicate significant differences in gaze behavior between the two groups.

Significant differences were found in the EGs of the experts and novices. To our knowledge, no previous studies have explained the mechanisms underlying these results. Qualitative interview data are useful for a better understanding of the observed quantitative results [18]. Therefore, a qualitative study was conducted in the second phase, in which the narratives of the parties involved were collected and analyzed to understand the quantitative results.

### Second phase: Qualitative research

#### Qualitative approach

This study employed a qualitative descriptive approach, which is based on naturalistic inquiry. By approaching the phenomenon of interest without modifying the immediate environment [44], minimizes theorizing. Additionally, it aims at a frank description of the phenomenon and is suitable for gaining knowledge in areas where little research has been conducted [45]. This study considered a qualitative descriptive approach to be suitable, as its aim was to frankly describe experts and novices’ perceptions of their gaze behavior, an area that had not been explored to date.

#### Data collection: Cued retrospective reporting

The three main methods for verbalizing cognitive processes during task performance are think-aloud reporting, retrospective reporting, and cued retrospective reporting [27, 46–48]. Think-aloud reporting involves participants verbalizing their thoughts and cognitive processes in real-time as they perform the task [27, 46–48]. The main issues with this method are that verbalization can interfere with task execution, and the speed of thinking is faster than speaking, leading to only a partial report of cognitive activities [27, 46–48]. Retrospective reporting involves participants recalling the thoughts and cognitive processes that occurred during task performance and verbalizing them afterward [27, 46–48]. Since this method relies on participants’ memory, there is a risk of generalization, interference, and forgetting, which may lead to post-hoc rationalizations, bias, or even fabrication [27, 46–48]. Cued retrospective reporting is a method where cues related to the task are provided after task completion to help participants recall and verbalize the cognitive processes they experienced during the task [27, 46–48]. This method is less susceptible to the effects of forgetting or fabrication compared to pure retrospective reporting, and it allows for more accurate verbalization of cognitive processes [27, 46–48]. Further, presenting eye-tracking data as cues, which reflect cognitive processes, enables participants to more precisely explain the “why” and “how” of their actions during the task [27, 46–48]. In fact, previous studies that used eye-tracking data as cues in cued retrospective reporting have qualitatively described the cognitive processes of expert clinicians during cardiopulmonary resuscitation and reported differences in cognitive approaches between medical students, residents, and physicians in electrocardiogram interpretation [18, 49].

This suggests that presenting experts and novices with their eye-tracking data as cues could provide qualitative insights into how they think and use eye movements during simulated medical interview. Such qualitative data could be useful in explaining the differences in eye movement behavior between experts and novices. To identify the cognitive processes reflected in eye movements, we conducted an interview using a combination of cued retrospective reporting and eye-tracking.

**Fig 5:**
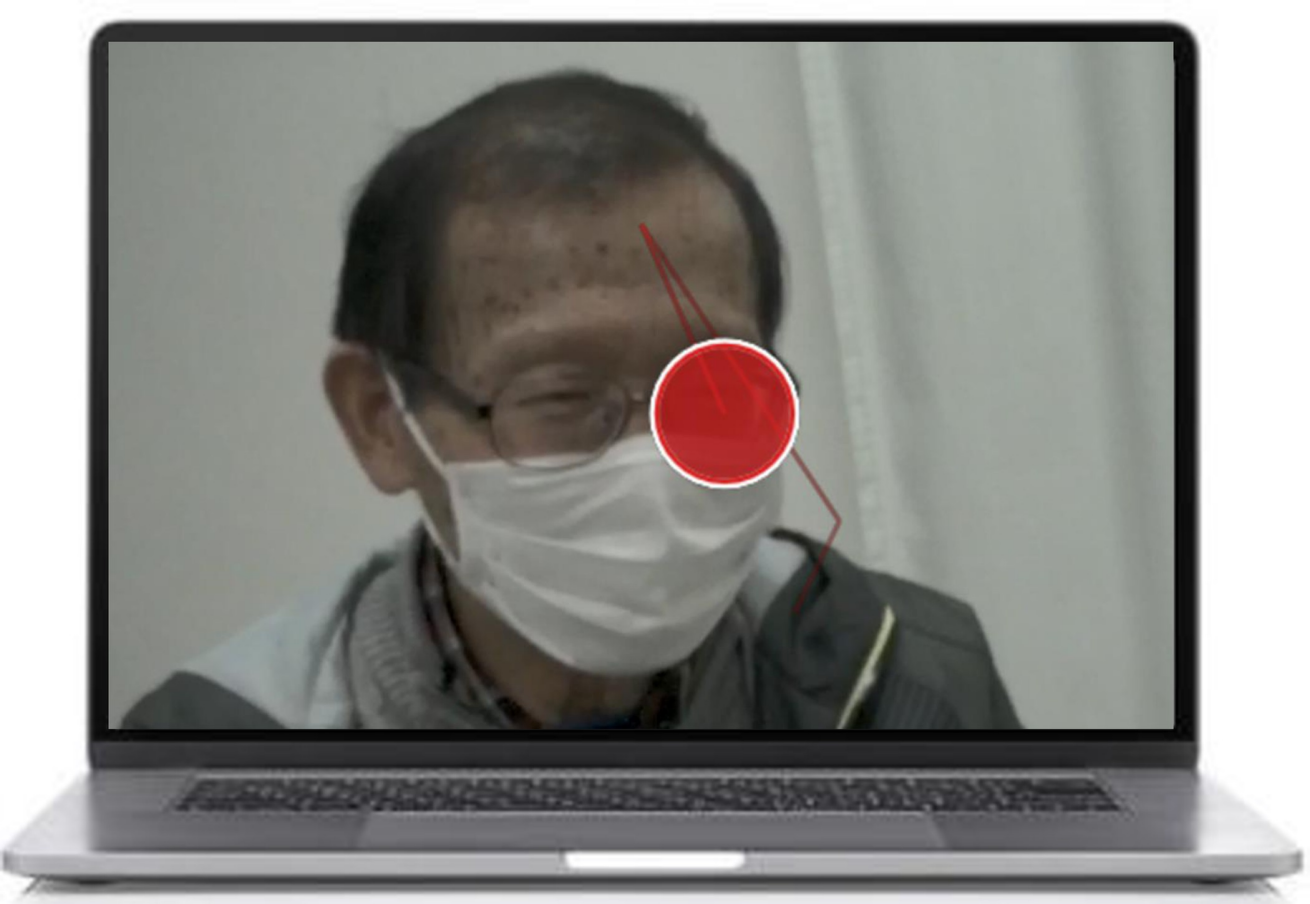
The paused image of the replayed EG video. The video of the simulated medical interview, recorded from the perspectives of both experts and novices, was played, and the playback was paused at the point when the experts’ and novices’ EG were displayed.

Qualitative data were collected by following the procedure described below. After completing the simulated medical interview, RY interviewed each expert and novice in an examination room, and with their consent, the interview was recorded using an IC recorder. The interview began with the playback of the video footage of the experts and novices’ gaze behavior during the medical interview, and each time their EG video footage was played, RY paused the video (Fig 5). They were then interviewed using a semi-structured interview guide comprising three questions: 1) Did you notice anything when you watched the video of your gaze behavior? 2) What did you think about when you looked into the SP’s eyes? and 3) Did you think about anything else when you looked into the SP’s eyes? Experts and novices retraced their cognition while watching a video of their gaze behavior and spoke freely in response to the questions.

To obtain as much detailed information as possible, RY asked additional questions depending on the content of their narratives, such as “Can you be more specific about what you just told me?” The average interview time was 23 minutes (10–54 minutes).

Additionally, informed consent for the publication of images was obtained from all participants whose images are presented in Figs 1, 2 and 5.

#### Qualitative analysis

All the recordings were transcribed verbatim into Japanese and used as data. The verbatim transcripts were quality-checked by RY, and then translated into English. RY and SK analyzed the data using the analytical procedure of Sadowski’s qualitative descriptive approach [45].

First, the data were read repeatedly to understand its overall content. Second, individual analyses were conducted for each participant. Narratives of participants’ cognition of their gaze behavior were extracted and coded so that no semantic content was lost. The codes were reviewed, and those with commonalities were aggregated into subcategories, and subcategories with commonalities were aggregated into categories. Disagreements between the two coders throughout the analysis process were resolved through discussions between MF and KX.

### Data trustworthiness

The following four criteria were identified [50] for assessing reliability of the data: credibility, transferability, dependability, and confirmability. Based on these criteria, to ensure prolonged involvement, the researchers participated in the activities of participants and tried to understand their thinking and behavioral patterns. Data analyses were shared with the research team, and an agreement was reached on data interpretation.

To ensure triangulation, we used multiple data sources, including interview data, video recordings of EG behavior during a simulated medical interview recorded from both expert and novice perspectives, and eye-tracking data. Based on these data, medical education researchers and health science researchers on the research team engaged in thorough discussions to enhance the credibility of this study. Member-checking was conducted with participants and supported as an appropriate representation of their perceptions of EG. The researchers maintained a consistent reflective stance throughout, to monitor their subjectivity and minimize their own influence on data collection and analyses.

### Qualitative results

#### Cognition when experts gaze into patients’ eyes during medical interview

Three categories and eight subcategories emerged from the experts’ qualitative data (Table 3). The three categories were: (1) **observing the patient to obtain clues for diagnosis**; (2) **being careful not to look too closely into the patients’ eyes**; and (3) **attempting to establish an ongoing relationship with the patients by using gaze behavior**. Each of these categories reflect the experts’ cognitive behavior. Looking into patients’ eyes can lead to physical findings and subjective information; which can lead to a diagnosis or build a relationship with patients; that can lead to further treatment. In the paragraphs that follow, categories are shown in **bold**, subcategories in “double quotes,” experts and novices’ narratives in *italics*.

**Table 3:** Overview of categories and subcategories about experts’ cognition.

Categories	Subcategories
	Confirming malnutrition from the dry skin on the patients’ faces
Observing the patients to obtain clues for diagnoses	Confirming microcirculation disorders from the patients’ facial color and pigmentation
	Confirming by checking the patients’ eyelids and face for edema
	Looking too much into the patients’ eyes can make them nervous
Being careful not to look too closely into the patients’ eyes	Looking too much into the patients’ eyes can make them uncomfortable
	Eye contact can be judged as too much eye gaze
Attempting to establish an ongoing relationship with the patients by using gaze behavior	Try to check the level of satisfaction from patients’ facial expressions
	We try to read the patients’ facial expressions

#### Observing the patients to obtain clues for diagnoses

All the experts explained that to make an accurate diagnosis and provide treatment, it is necessary to obtain multiple findings—one of which is to observe the patients and obtain physical findings. Therefore, they said that they observed the eyes and faces of patients, who were not covered by masks for “confirming malnutrition from the dry skin on the patients’ faces” and “confirming microcirculation disorders from the patients’ facial color and pigmentation.” They cognized, observing the findings as doing something naturally, rather than consciously.

*Some prescriptions come immediately to mind when patients say that their chief complaint is nocturia. Putting that aside, when I medically examine the patient, I notice the dry skin on the face, which suggests mild malnutrition. After observing the findings and making a differential diagnosis, I decided on a prescription medicine*. (Expert-G)

*Firstly*, *the patient’s chief complaint was nocturia*, *Although*, *I considered several prescription drugs*, *I needed to examine the patient from various aspects to arrive at multifaceted findings*. *I thought that the patient’s having many facial spots made the face look dark; so*, *I felt that the patient might have microcirculation disorders*. (Expert-B)

Regarding wanting to prescribe the best medication for patients, the experts stressed the need to know if, and to what extent, symptoms were present by observation, as patients may not be aware of their symptoms. They explained that as edema can be perceived differently depending on sex “confirming by checking the patient’s eyelids and face for edema” is important.

*I don’ t make prescription decisions based on the chief complaint alone; so, even if a patient states on the medical questionnaire that there’s no edema, I look to see if there really isn’t. I think that women probably look into the mirror a lot, whereas, I don’t think men look as much. I think it is difficult for them to notice that their eyelids or face are edematous. So, I check by looking at their eyes and face.* (Expert-A)

#### Being careful not to look too closely into the patients’ eyes

Most experts emphasized the importance of listening to patients, allowing them to feel comfortable discussing their symptoms and thoughts about their chief complaint, which provides essential clues for an accurate diagnosis. From their past clinical practice, the experts said that “looking too much into the patients’ eyes can make them nervous” and “looking too much into patients’ eyes can make them uncomfortable.” They explained that daring to not look into the patients’ eyes too much could elicit patients’ chief complaints.

*I am very careful not to look too much into the patients’ eyes, so that they can relax. If I look too much, the patients get nervous, and if they can’t say what they want to say, they don’t get better. I think if I don’t look into the patient’s eyes too much, they won’t get nervous*. (Expert-E)

*I try to make sure that the patients feel that they have been able to divulge everything that is bothering them*, *rather than their feeling gloomy because they can’t say what they want to say*. *The main complaint can be one of the deciding factors*. *When I listen*, *I try not to look into the patients’ eyes or face too much because I think it makes them feel uncomfortable*. (Expert-F)

The expert, who said that consultations cannot begin without listening to patients, explained that although eye contact is generally emphasized when listening to patients, in clinical settings with diverse medical needs, “eye contact can be judged as too much eye gaze.”

*First, listen to the patients. Tell them to speak freely by asking an open-ended question: How are you feeling? From a textbook point of view, it is important to make eye contact, but some patients may feel that you are looking at them too much. There are also patients who are a bit down, and have different ways of thinking*. (Expert C)

#### Attempting to establish an ongoing relationship with the patients by using gaze behavior

The experts who emphasized non-verbal communication with patients said that they used gaze behavior to avoid looking too much into the patients’ eyes, as first-time patients are often nervous. They explained that it is important to make patients feel comfortable during the first visit, and advised, “try to check the level of satisfaction from patients’ facial expression,” leading to continued treatment.

*I really think it would be good if we could provide non-verbal care, such as gaze behavior. First-time patients are nervous at first, aren’t they? However, when I use gaze behavior, the patients gradually relax, and at the end of the examination, leave with smiles on their faces. So, when that happens, I know that it went well.* (Expert-E)

The experts who determined the need for continued treatment based on the patients’ age and chief complaints said, “We try to read the patients’ facial expressions” at the first meeting because a relationship has not yet been established. They explained that it is important to tell from subtle changes in the patients’ facial expressions whether the conversation should be continued. They believed that careful observation of such non-verbal cues was the key to building a continuous relationship.

*A medical interview is not something where I ask patients questions, and patients just answer yes or no. Especially since it’s the first consultation, and I don’t even know the patient. It is also about building a relationship. Therefore, I must observe patients’ faces and listen to the way they speak, to see if they look uncomfortable or if they are satisfied with the conversation. It is not a good thing to meddle with first-time patients’ personal affairs, as there are some things that patients don’t want to touch upon, aren’t there? The patients are willing to talk about them to a certain extent, once a relationship has been established. Considering that nocturia gets worse every year, I thought it would be better to consider that the relationship could be long-term, and ongoing treatment is needed*. (Expert-A)

#### Cognition when novices gaze into patients’ eyes during medical interview

Three categories and eight subcategories emerged from the novices’ qualitative data (Table 4). Each category reflects the novices’ cognition, that looking into patients’ eyes is more about building personal relationships and facilitating communication, than obtaining the physical findings necessary for diagnoses. The three categories are: (1) **looking at patients and trying to increase their satisfaction**; (2) **confirming changes in patients’ expressions and trying to build a relationship in the here and now**; and (3) utilizing their gaze-related experience and knowledge for their eye behavior.

**Table 4:** Overview of categories and subcategories about novices’ cognition.

Categories	Subcategories
	Consciously try to look at patients and make them feel at ease
Looking at patients and trying to increase their satisfaction	Look at patients and try to build a relationship with them
	Look at patients and create an atmosphere where it is easy to talk to them
Confirming changes in patients’ expressions and trying to build a relationship	Careful to look for changes in patients’ facial expressions
	Trying to detect changes in patients’ sense of tension
Utilizing their gaze-related experience and knowledge for their eye behavior	Taught to look into their patients’ eyes and listen to what they had to say
	Many of these doctors typed in the chart without looking at the patient at all
	A family member complained about a doctor not looking at the patient

#### Looking at patients and trying to increase their satisfaction

All the novices stated that by looking into the patients’ eyes, they were trying to increase patient satisfaction in terms of reassurance, relationship building, and smooth communication. Most novices stated that their reason for looking at patients was to increase satisfaction, rather than to obtain findings that would lead to a diagnosis.

Novices who were concerned that, as medical students, they might make patients anxious by conducting a medical interview explained that they “consciously try to look at patients and make them feel at ease,” so that they would not feel anxious.

*Because I am still a medical student, I am careful not to make patients too anxious. I think it’s important to look at their eyes and faces as much as possible, so that they don’t feel anxious. Therefore, I’m always careful*. (Novice-A)

Novices who emphasized patients’ personhood, saw person-to-person and doctor–patient relationships as incompatible, and because of this desire to focus on the person-to-person relationship, a novice explained: “look at patients and try to build a relationship with them.”

*I look into the eyes and faces of patients because I have a strong desire to treat them as human beings. Of course, it is important to look at their affected parts and arrive at findings, but before that, when I see them in front of me, I prefer a person-to-person relationship, rather than a doctor-to-patient relationship*. (Novice G)

A novice, who spoke about wanting to first provide the patient with medicine, believed that using eye contact to send non-verbal signals about being interested in the patient leads to smoother communication. This novice explained about attempts to “look at patients and create an atmosphere where it is easy to talk to them.”

*Looking into the patient’s eyes was not so much about trying to get a diagnosis, but more about building a relationship, which I think is a strong image for me. By looking at the patient, I thought it would be good if I could create an atmosphere, where the patient would be willing to talk about anything. It depends on individual patients, but I think most of them want to be listened to; so, I hope to create an atmosphere where they can talk about what they want to talk about*. (Novice B)

#### Confirming changes in patients’ expressions and trying to build a relationship

A novice, who valued interactions with patients, explained about refraining from asking new patients detailed questions and being “careful to look for changes in patients’ facial expressions,” so as not to delve too deeply into what they do not want to talk about. Additionally, the novice believed that such considerations were important for building relationships in the here and now.

*When I say something, to better understand the patients’ reactions—what they want and do not want to say—I want to communicate with them as much as possible, watch their facial expressions, and so on*. (Novice-A)

Novices emphasized the importance of establishing a rapport with patients. A novice shared, “trying to detect changes in patients’ sense of tension” through non-verbal cues, such as their mood and changes in facial expression. Other novices saw it as establishing a relationship in the present moment, when they could confirm that the patients’ tensions were relieved.

*At first, while we were checking the patient’s name, I think there was still some tension between us, but after a minute or two, I personally felt that the atmosphere gradually relaxed. As we talked, the patient seemed to open more and more. I think I was able to talk relatively well from around the middle of the interview.* (Novice B)

#### Utilizing their gaze-related experience and knowledge for their eye behavior

All the novices reflected on one of the following three types of knowledge and experience related to gaze behavior: knowledge taught in lectures and practical training, experience of independent learning in practical training, or experience outside of lectures and practical training. Through such knowledge and experience, they recognized that looking at the patients was necessary for an appropriate medical interview with them.

Novices who mentioned that eye contact knowledge were taught in lectures and practical training, also talked about gaining knowledge from OSCE video materials and practical training. They were “taught to look into their patients’ eyes and listen to what they had to say” by their supervisors. They explained that by using such knowledge, they tried to look into their patients’ eyes

*In the OSCE video, we were told to look into the patients’ eyes*. (Novice-E)

*I was taught during the placement session that it is better to look at the patients’ eyes and face when you meet them for the first time*, *so that you can communicate well with them*. (Novice-C)

Novices, who cited gaze behavior as an experience that they independently learned during their practice, said that when they had sat in consultations with supervising doctors, they had observed that “many of these doctors typed in the chart without looking at the patient at all.” A novice explained that she was conscious of looking her patients in the eye because of having a good example of who not to be.

*When I looked at the teachers in practice, there were quite a few who were looking at the medical records all the time. I couldn’t help but be bothered. Therefore, I want to look at the patients’ eyes and face. I want to communicate with patients while looking at their eyes and facial expressions. I think it’s OK, even if my notes in the medical chart are simple, as I can correct them later.* (Novice-E)

A novice described an instance, in which, “a family member complained about a doctor not looking at the patient,” as a private experience of gaze behavior outside of lectures and practice. Based on the negative experiences of such close family members and familiar others, the novice explained about being careful to look at the patients to avoid making them feel uncomfortable.

*I wanted to be a physician from the point of view of patients. My grandfather used to complain that he didn’t like physicians who didn’t see patients at all. I don’t want to be a physician, who says, “Wait a minute, I’m writing a medical chart now,” when a patient comes into the examination room and tries to talk to me.* (Novice-F)

## Discussion

Previous studies on experts’ gaze measurement have primarily focused on diagnostic imaging, such as X-rays or MRI [15–18], and surgical or therapeutic situations [19, 20]. Little research has been conducted on the medical interview, which accounts for 80% of diagnoses [1, 2]. This study is the first to quantitatively evaluate experts’ gaze behavior during a simulated medical interview and qualitatively explore the cognition closely involved with gaze behavior. By doing so, it aims to visualize the dual function of experts’ gaze behavior—signal transmission and information gathering—providing valuable insights for medical education that had not been previously investigated.

In the first phase, the gaze behavior of experts and novices was quantitatively evaluated during the simulated medical interview, showing that experts looked at the SP’s eyes less frequently than novices. Previous studies have also shown that experts do not look at specific areas more frequently than novices when collecting visual information during diagnostic imaging [15–18] or during treatment and procedures [19, 20]. Taken together, these findings suggest that across different phases of medical practice—whether in a medical interview, diagnostic imaging, or treatment—experts possess the ability to rapidly identify key areas and efficiently gather visual information.

In the second phase, qualitative data on the cognition of experts and novices were collected to gain a deeper understanding of the quantitative results. This study used five gaze behavior types (SP’s eyes, face, body trunk, medical chart, and medical questionnaire); among them, the only type that showed a significant difference between experts and novices was gaze toward the SP’s eyes. Both experts and novices recognized that they looked at the patient’s eyes to read facial expressions. Concerning facial recognition, Westerners focus on the mouth, while Japanese individuals focus on the eyes [51]. Additionally, older adults may look at the eyes less frequently than younger adults when identifying emotions, whereas younger adults tend to look more at the eyes [52]. Participants were Japanese, with an average age of 51.9 years for experts and 27.6 years for novices. Considering these factors, both experts and novices looked at the patient’s eyes owing to their cultural background; however, the significant difference in the frequency of EG may be attributed to age-related differences in emotional processing. The combination of cultural background and age-related differences in emotion processing likely resulted in the significant difference in gaze behavior toward the SP’s eyes, among the five gaze behavior types.

Further, two differences were observed between experts and novices regarding their cognition of gazing at the SP’s eyes. The first difference lies in the cognitive difference regarding how gaze behavior as signal transmission is received by the patient. Experts recognized that gazing at the patient’s eyes could cause tension or discomfort, thus they gazed at the eyes less frequently. In contrast, novices recognized that gazing at the patient’s eyes would improve patient satisfaction, so they directed their gaze more frequently. Individuals perceive eye contact as being established when another person’s gaze is directed toward a part of their face [53]. The more frequently experts and novices gazed at the patient’s eyes, the more likely the patient was to perceive that eye contact was made. In general, individuals who make eye contact are evaluated as being more likable, intelligent, and trustworthy [54]. However, in Japanese culture, individuals who make eye contact are often perceived as being angry, intimidating, or uncomfortable [54], and avoiding direct eye contact is often seen as a sign of respect or humility [55]. This cultural background may have related the gaze behavior of experts as signal transmission, resulting in them avoiding direct eye contact with the patient. In clinical settings, experts may feel that reducing direct eye contact helps the patient relax and facilitates building a trusting relationship. However, novices, with less clinical experience, may base their gaze behavior on a general understanding of eye contact as a form of signal transmission, leading them to gaze at the patient’s eyes more frequently. In recent years, Western values and culture have increasingly influenced Japan, and medical textbooks now encourage making eye contact with patients. As noted earlier, the experts in this study were older, while the novices were younger, suggesting that generational differences in the influence of Western culture may also have contributed to the cognitive differences in how gaze behavior as signal transmission was perceived by patients.

While eye contact between physicians and patients has been emphasized [21–24], the importance of clinical practice and medical education that consider cultural differences in gaze behavior, including eye contact, has not been sufficiently clarified [56]. Failure to address cultural differences may lead to misdiagnosis, medical errors, and suboptimal treatment outcomes [57]. In this study, Japanese experts explained that they avoided frequent eye contact with patients to prevent causing tension or discomfort. These results suggest that considering cultural differences in gaze behavior is important both in clinical practice and in medical education. For healthcare providers, it is crucial to understand and respect patients’ cultural background, rather than relying solely on their own cultural perspective, as this can help prevent medical errors and lead to optimal treatment outcomes. Such a perspective is essential in medical education curricula to promote appropriate communication during encounters with culturally diverse patients.

Another difference lies in the cognition associated with gaze behavior as information gathering necessary for diagnosis. Experts mentioned that they looked at the patient’s eyes to observe abnormal findings. Although they gazed less frequently than novices, they observed a wide range of visual cues, such as eyelid or facial edema and pigmentation. Experts gather information from a broad visual field [58], quickly making an overall assessment of the image and then identifying potential abnormal findings [59]. This suggests that experts have distinct cognitive processes in which they observe diagnostic areas while maintaining an overall perspective, collecting visual information without being limited to specific areas. In contrast, novices reported looking at the patient’s eyes not to observe diagnostic findings but to provide patient-centered care, build relationships, and facilitate smooth communication; thus, they did so more frequently. Observers can typically only see three to four objects at a time [60]. In situations requiring the processing of large amounts of visual information and focused attention, important clinical information may be missed, even when it is clearly within the field of view [61]. Additionally, junior and prospective medical students tend to prioritize empathy, patient-centered care, and communication skills over clinical competence and knowledge [62]. For novices, a medical interview with an SP they are meeting for the first time may be an overloaded situation, requiring them to focus and process large amounts of visual information, likely causing some confusion and frustration. In such physically and mentally stressful circumstances, even senior medical students with clinical training experience may, like junior students, struggle to fully execute the dual function of gaze behavior—signal transmission and information gathering—and may prioritize signal transmission, focusing more on care.

In the early stages of clinical training, novices are prone to stress and may not fully execute the dual function of gaze behavior (signal transmission and information gathering). In this study, novices did not mention observing the area around the face for diagnosis or information gathering, suggesting that they could be unaware of the need to balance information gathering and signal transmission. Therefore, it is important for instructors to remind novices at the beginning of clinical training that effectively utilizing the dual function of gaze behavior—signal transmission and information gathering—can help achieve the goals of the medical interview: gathering information necessary for diagnosis and treatment as well as building relationships with patients. For instance, one approach is for experts to demonstrate their gaze behavior while verbally explaining the process of interpretation [63], which can enhance medical students’ visual search and symptom interpretation skills [64]. By presenting their own gaze behavior and accompanying cognitive processes, experts can help novices deepen their interest in and understanding of gaze behavior as a means of visual information gathering. Additionally, novices should be made aware that they tend to prioritize signal transmission in their gaze behavior, highlighting the importance of training to develop a broader visual perspective, like experts, to search for abnormal findings necessary for diagnosis during the medical interview.

Traditionally, eye contact between physicians and patients has been emphasized; however, this study revealed that experts use the act of looking at the patient’s eyes to build rapport and gather the necessary visual information for diagnosis from a broader perspective. While gazing at the patient’s eyes is important during a medical interview, experts recognize their gaze behavior as serving the dual function of signal transmission and information gathering, whereas novices only recognized it as signal transmission. These results suggest that experts’ gaze behavior, beyond just eye contact, and their awareness of it, hold key importance in clinical practice and medical education, offering new perspectives for research and training on gaze behavior.

### Study limitations

This study had some limitations. First, using only one SP may have highlighted individual differences between experts and novices. We accounted for individual differences among participants by using a linear mixed model to eliminate random effects. However, following the speed-interaction methodology of Hoffmann et al. [14], we believe that conducting simulated consultations with multiple SPs of different ages, sexes, symptoms, and stages of illness would strengthen the validity of the results and enhance statistical power through repeated measurements. Therefore, verification with multiple SPs and diverse scenarios remains a challenge for future research.

Second, the experts were physicians; if they had been doctors from other specialties, such as surgeons, psychiatrists, or pediatricians, their gaze behavior and cognition may have differed from the current findings. However, as this study focused on the comparison between experts and novices, such differences in specialties are not believed to have directly impacted its results.

Third, participants’ medical knowledge, skills, prior experiences, and personal values, which they have developed over time, may have influenced their gaze behavior toward the SP’s eyes and their cognition.

Fourth, this study did not investigate the relationship between eye-tracking data and interview data using statistical methods, making it difficult to assert that there is a significant relationship between the two. In future research, it is necessary to incorporate quantitative methods to statistically examine the relationship between eye-tracking data and interview data. Additionally, conducting studies to predict psychological and behavioral outcomes, such as patient satisfaction and trust in physicians, based on physicians’ gaze behavior toward patients is also considered an important challenge.

Fifth, because we adopted a mixed methods approach combining eye-tracking (quantitative research) and cued retrospective reporting (qualitative research), it was difficult to determine the sample size using a power analysis. Although a power analysis was applicable to the diverse experiences and perspectives of participants in the qualitative research, we judged that the size calculation was inappropriate. Therefore, referring to prior research [18, 65], we set the sample size to 17 participants based on a comprehensive judgment. However, the inability to utilize a power analysis is a limitation. Sixth, to enhance internal validity, cued retrospective reporting was used to reinforce participants’ memory when verbalizing their awareness of gaze behavior. However, some explanations regarding their awareness of gaze behavior may have been post-rationalized. To address this, measures such as triangulation and member-checking were implemented to increase reliability, but completely eliminating post-rationalization remains a challenge. Future studies could collect data on confounding factors, such as participants’ age, sex, and experience level, in advance and use statistical methods to control these variables to further enhance internal validity.

Seventh, to enhance external validity, we used an outpatient examination room, where experts conduct their daily practice and novices have experienced an initial patient interview during their clinical training. This helped increase the realism of the simulated medical interview. However, since participants were aware that this was a simulation, some of their behavior may have been intentionally controlled. This is particularly true for novices, who may have experienced heightened anxiety, potentially limiting their cognition related to gathering visual information. Further, real patient interactions may involve more unpredictable emotional and behavioral responses that differ from those in a simulation. Therefore, it cannot be ruled out that their behavior differed from what would occur in an actual clinical setting. In further studies, masking techniques should be implemented to ensure that participants are unaware of the simulation, allowing for more natural behavior. Additionally, comparing and validating a medical interview using real patients and SPs should enhance external validity.

Finally, cultural differences exist in the way eye contact is perceived. The experts and novices in this study were Japanese, and it cannot be ruled out that the Japanese-specific perception of eye contact avoidance being positive, may have influenced gaze behavior and cognition. Thus, the generalizability of the results is limited. In addition, as we used a cross-sectional design, the gaze behavior and cognition of experts and novices were outside its scope. Future studies should increase the sample size and number of SPs, as well as consider the specialization of doctors, symptoms of SPs, and their disease stage. Additionally, a longitudinal study on the factors that influence the gaze behavior of experts and novices, and the process of cognitive mastery, would provide knowledge that could be fed back to learners at different developmental stages.

## Conclusion

This study quantitatively evaluated the gaze behavior of experts and novices in a simulated medical interview and qualitatively described their cognitive processes. Experts looked at the SP’s eyes less frequently than novices, as experts recognized this as important for gathering diagnostic information and building rapport, while also acknowledging that it can cause discomfort. While novices did not mention information gathering, they recognized that gazing at the eyes was important for building rapport. These differences in cognition were related to the differences in gaze behavior between the two groups. Although traditional approaches have emphasized gazing at the eyes, the current findings provide valuable insights into the instruction of gaze behavior beyond eye contact, enhancing novices’ learning effectiveness. Future research should longitudinally investigate the factors that influence the acquisition of gaze behavior in both experts and novices.
